# Comparison of Expressive Spoken Language Skills in Children With Cochlear Implants and Children With Typical Hearing

**DOI:** 10.3389/fpsyg.2020.01405

**Published:** 2020-07-15

**Authors:** Michaela Socher, Rachel Jane Ellis, Malin Wass, Björn Lyxell

**Affiliations:** ^1^Swedish Institute of Disability Research, Linköping University, Linköping, Sweden; ^2^Department of Business Administration, Technology and Social Sciences, Luleå University of Technology, Luleå, Sweden; ^3^Special Needs Education, University of Oslo, Oslo, Norway

**Keywords:** expressive grammar, expressive vocabulary, working memory, non-verbal intelligence, cochlear implant, children

## Abstract

When children start formal education, they are expected to be able to express complex thoughts. However, in order to do so, they need to be able to use both complex grammatical structures and a variety of words. One group that is at risk of having a delay in terms of their expressive language ability is children with cochlear implants (CI). In this study, we evaluated whether children with CI perform comparably to children with typical hearing (TH) on a standard expressive spoken grammar and a standard expressive spoken vocabulary task when the groups were matched on non-verbal intelligence and working memory capacity. It was found that the children with CI in this study performed more poorly on a standard expressive spoken vocabulary task but not on a standard expressive spoken grammar task when compared to the children with TH. Differences in terms of expressive spoken vocabulary do not seem to be explained by differences in cognitive ability. In addition, the variation in terms of expressive spoken language ability was larger in the children with CI compared to the children with TH. This might be explained by additional confounding factors, like the time of language deprivation or by a greater influence of cognitive differences for the acquisition of spoken language for children with CI.

## Introduction

When children reach school-age, they are expected to use their native language with ease. They should be able to use both complex grammatical structures and a large variety of words. This language proficiency is necessary to express complex thoughts, beliefs, and desires, which is essential to participate in learning and social activities in school. In addition, grammatical competence (Bishop and Adams, 1990) and vocabulary skills (Biemiller, 2003) have been linked to literacy development and expressive grammar has been found to be important for peer social behavior in typically developing kindergarten children (NICHD, 2001). However, one group that is at risk of not being able to use language in an age-appropriate way when starting formal education is deaf and hard of hearing children with cochlear implants (CI) (Geers et al., 2009). In this study, we aim to evaluate whether differences in expressive spoken language ability between children with CI and children with typical hearing (TH) can be explained by cognitive differences, and whether individual differences in cognitive abilities have a larger influence on the expressive spoken language ability of children with CI when compared to children with TH.

Children with CI are at risk of performing more poorly on expressive spoken language tasks compared to peers with TH (Nicholas and Geers, 2007; Duchesne et al., 2009; Inscoe et al., 2009; Nott et al., 2009; Boons et al., 2012, 2013; Lund, 2016). Cupples et al. (2018) found that children with CI or hearing aids (HA) perform approximately one SD below children with TH on tasks measuring expressive spoken language. One explanation for this is that children with CI are likely to experience a time of language deprivation early in life. A large proportion of children with CI are born to hearing parents (Mitchell and Karchmer, 2004). As hearing parents are unlikely to be fluent signers, the children are likely to have limited access to language before receiving their implant. Another explanation is that hearing with a CI leads to a distorted input signal and it might, therefore, require more cognitive resources to acquire and use spoken language using a CI in comparison to when having TH. This is in accordance with results by Inscoe et al. (2009). The authors found that a larger percentage of children with CI do not have expressive spoken language skills at their hearing age level (time since they received their CI). Additionally, cognitive functions like non-verbal intelligence (Geers et al., 2003a; Tobey et al., 2003; Holt and Kirk, 2005) and working memory (Willstedt-Svensson et al., 2004) have been found to be associated with expressive spoken language skills in children with CI. van Wieringen and Wouters (2015) argued that differences in executive function skills, like working memory, might be one explanation for the variation in terms of spoken language outcome seen in children with CI. However, non-verbal intelligence (van der Schuit et al., 2011) and working memory (Newbury et al., 2015) are also associated with the spoken language ability of children with TH. It could be argued that while cognitive skills are associated with spoken language ability, both in children with CI and children with TH, the influence of cognitive skills on spoken language outcome might be larger in children with CI due to the distorted input signal. One indication for this is the large variation in terms of expressive spoken language in children with CI (Chilosi et al., 2013; Hess et al., 2014), which might be explained by a larger influence of individual differences (for example, in terms of cognitive ability). However, neither Chilosi et al. (2013) nor Hess et al. (2014) have compared this variation to a matched group of children with TH. It is therefore unclear if the variance seen in children with CI is larger than in the typically developing population.

Non-verbal intelligence (Geers et al., 2003a; Tobey et al., 2003; Holt and Kirk, 2005) and working memory (Kronenberger et al., 2013, 2014b) have been found to influence spoken language ability in children with CI and children with TH (van der Schuit et al., 2011; Newbury et al., 2015) but children with CI have also been found to be at risk of performing more poorly on non-verbal intelligence (Cejas et al., 2018) and working memory tasks (Harris et al., 2013; Kronenberger et al., 2014b) compared to children with TH. Cejas et al. (2018) found that children with CI have non-verbal intelligence scores in the normal range, but still perform significantly more poorly than children with TH even after controlling for maternal education. More importantly, verbal working memory ability has been found to be significantly lower in children with CI even if they are compared to children with TH matched on non-verbal intelligence (Kronenberger et al., 2014b). Conway et al. (2009) assume that auditory deprivation leads to disturbed sequencing ability due to the absence of auditory scaffolding. This disturbed sequencing ability might in turn lead to lower verbal working memory skills in children with CI. von Koss Torkildsen et al. (2018) argue against this claim. They found no lower performance of children with CI in terms of sequential learning. They argued that studies finding differences are often using tasks which encourage verbal rehearsal strategies. One additional reason for differences in both non-verbal intelligence and working memory might be a sample selection bias. The children with TH that take part in studies as control groups might be children with a higher cognitive ability than the general population, while the children with CI might be more diverse. Parents might be more willing to let their child participate in the control group of a scientific study if they assume their child will “do well.” This has not yet been evaluated but it seems reasonable to assume that this might be the case given that sample selection bias has been found in other research areas (Larzelere et al., 2004; Roe et al., 2009). An additional factor leading to differences between children with CI and children with TH in terms of cognitive ability could also be a high proportion of additional disabilities, especially developmental delays (Birman et al., 2012) and neuropsychological problems (Kronenberger et al., 2014a) in children with CI. Being deaf or hard of hearing seems to delay the diagnosis of developmental disorders (Roper et al., 2003; Mandell et al., 2005). Therefore, a large percentage of these disabilities might not be detected in children with CI until they are older but might still influence the child’s cognitive performance during the time of testing.

Matching is essential for meaningful group comparison. It reduces the influence of confounding factors and thereby limits the number of possible interpretations of a result (Facon et al., 2011). Two factors associated with expressive spoken language ability are verbal working memory (Willstedt-Svensson et al., 2004; Newbury et al., 2015) and non-verbal intelligence (van der Schuit et al., 2011; Cupples et al., 2018). It is important to control for these abilities when comparing the expressive spoken language ability of children with CI and children with TH. If the comparison groups differ on verbal working memory ability and/or non-verbal intelligence, differences in expressive spoken language ability might be explained by underlying cognitive differences between the groups. For non-verbal intelligence, a procedure often chosen is to exclude children with a non-verbal IQ below 70 or 80 (Nicholas and Geers, 2007; Duchesne et al., 2009; Geers et al., 2009; Boons et al., 2012, 2013; Chilosi et al., 2013). However, this does not prevent differences between comparison groups in terms of non-verbal IQ. The mean and distribution of non-verbal IQ scores could still be statistically different. Facon et al. (2011) emphasized that it is important to not only match groups on mean but also on SD and that alpha levels need to be increased, preferably to 0.5 or larger, to ensure the groups are actually similar in the matched skills. According to Frick’s categories, matching is only ensured if the *p* of the comparison is 0.65 or larger (Frick, 1995; Mervis and Klein-Tasman, 2004). This means, that non-significant difference between groups in terms of non-verbal intelligence like reported by Nittrouer et al. (2014) does not mean that groups can be considered to be matched on this skill. If groups are not matched in terms of non-verbal intelligence, this might lead to differences in the mean and distribution in terms of spoken language, as non-verbal IQ has been found to be associated with spoken language in children with CI (Geers et al., 2003a, 2007; Holt and Kirk, 2005; Kronenberger et al., 2014b; Cupples et al., 2018) and children with TH (van der Schuit et al., 2011). Furthermore, no study has matched children with CI and children with TH on verbal working memory when comparing their expressive spoken language ability. However, verbal working memory has been found to be associated with spoken language in both children with CI (Willstedt-Svensson et al., 2004; Kronenberger et al., 2013, 2014b) and children with TH (Newbury et al., 2015). This means that even if comparison groups are matched on non-verbal IQ, differences in spoken language ability in terms of average performance and degree of variability might be explained by differences in working memory.

The aim of the current study is to evaluate whether differences in spoken expressive language in terms of average performance, like those found by, for example, Boons et al. (2013), and variance, like found, for example, by Hess et al. (2014) are still observed when comparing children with CI to children with TH matched on non-verbal intelligence and verbal working memory. The results will allow us to examine whether differences in expressive spoken language between children with CI and children with TH can be explained by differences in cognitive abilities. If this is the case, we would expect to find no differences in terms of average performance on the expressive spoken language tasks between groups matched on cognitive abilities. Similarly, we would expect to find no differences in terms of variation in expressive spoken language if these variations are mostly due to individual differences in non-verbal intelligence and verbal working memory.

## Materials and Methods

### Participants

Fifty-five children participated in the study. Seventeen of them had CIs and 38 were typically hearing (TH). A consent form was signed by the children’s caregivers. Both the children and the caregivers were told that they could drop out of the study without giving a reason. The study was approved by the local Research Ethics Review Committee in Linköping (Ref: 2015/308-31). All children received stickers for their participation.

Because of the different age distributions in the groups, it was decided to use scaled scores for the analysis of the expressive spoken language skills. The tests used to assess non-verbal intelligence and working memory are listed in the Material section. The statistical tests used to assess the matching were chosen depending on whether the data was normally distributed (Levene’s test and *t*-test) or not (Fligner-Killeen test and Mann-Whitney U test). Descriptive data regarding the participant groups are presented in Table 1. The children were individually matched in terms of their verbal working memory performance. For three children with CI, no child with TH could be matched in terms of verbal working memory performance and these three children have been excluded from the analysis. In addition, to improve the matching on non-verbal working memory, three of the children with TH were replaced with children not matched for verbal working memory (difference between child with CI and child with TH: 0.5, 1.5, and 2 points) but who were matched on non-verbal working memory. This led to a sample of 14 children with CI and 14 children with TH. The groups were matched (Frick, 1995; Facon, et al., 2011) in terms of verbal working memory, both on SD, *F*(1,26) = 0.085, *p* = 0.773, and mean, *t*(26) = -0.242, *p* = 0.811, *d* = 0.09. The groups were matched in terms of non-verbal intelligence, both on SD, *F*(1,26) = 0.007, *p* = 0.933, and mean, *t*(26) = -0.380, *p* = 0.707, *d* = 0.144. The groups matched in terms of non-verbal working memory, both on variance, *χ2* = 0.058, *p* = 0.810, and median, *U* = 88.5, *p* = 0.650, *r* = 0.090. The groups were found to differ significantly in terms of age distribution, *F*(1,26) = 10.526, *p* = 0.003, but not in mean age, *t*(17.133) = 0.817, *p* = 0.425, *d* = 0.308.

**Table 1:** The descriptive data for the children with CI and the children with TH.

Group	Age range	Average age	Female/Male	Unilateral/Bilateral CIs	Age at implantation range	Age at implantation mean and SD	Language
CI	5;6–8;2	6;8 (11)	9/5	2/12	0;5–5;6	23 (20) months	oral: 6 oral + sign support: 6 sign-language + oral language: 2
TH	5;7–7;1	6;5 (4.5)	7/7	-	-	-	oral: 14

The data concerning age at implantation and language use were collected by means of a questionnaire filled out by the caregivers.

The results from the non-verbal intelligence task have been reported in another study about pragmatic language ability (Socher et al., 2019). Missing data and differences in matching procedure led to the differences in terms of the sample include here and in the study about pragmatic language ability.

### Material

Expressive spoken language was tested by evaluating the children’s expressive spoken grammar and expressive spoken vocabulary skills. In addition, non-verbal intelligence, complex verbal working memory capacity, and non-verbal working memory were assessed and used to match the groups. All tests were administered in Swedish.

#### Expressive Spoken Vocabulary

To assess the expressive spoken vocabulary skills of the children, the expressive spoken vocabulary task from the Clinical Evaluation of Language Fundamentals IV (CELF-IV) battery (Semel et al., 2013) was used. In this task, the child was asked to name pictures. It started with a demonstration trial, and then two practice trials followed. The main task included 20 test trials. For the age group tested in the current study, the task started with trial three, leading to 18 test trials in total. If the child was unable to answer either test trial three or four correctly, the previous trials were presented in reverse order until the child had one point for two trials in a row. The child received one point for every correctly named picture. If the child was unable to name four pictures in a row, the task was terminated. The maximum amount of points a child could get was 20. The raw score was converted to the scaled score (Semel et al., 2013) and adjusted for age, for each individual child.

#### Expressive Spoken Grammar

The Formulate Sentences task from the CELF-IV battery (Semel et al., 2013) was used to measure expressive spoken grammar. In this task, the child saw a picture and was asked to describe the picture using a target word. The test consisted of a demonstration trial, two practice trials, and 22 test trials. The test was terminated if the child got 0 points for four sentences in a row. The child got two points for the sentence if it was a complete, grammatically, and syntactically correct sentence that included the target word(s) and fitted logically to the picture. The child got one point for a sentence if it was a whole sentence including the target word(s), logically fitting to the picture and had a maximum of two syntactical or grammatical errors, or a maximum of one conjunction or article missing. The child got 0 points for a sentence if the sentence did not meet the criteria for one or two points. The maximum amount of points a child could get for this test was 44. The raw score was converted to the scaled score (Semel et al., 2013) and adjusted for age, for each individual child.

#### Non-verbal Intelligence

The Matrices test from the Wechsler Nonverbal Scale of Ability (WNV) test battery was used (Wechsler and Naglieri, 2007). This test consisted of a demonstration trial, three practice trials, and 41 test trials. The first six test trials were bypassed (Wechsler and Naglieri, 2007). For each trial, the child saw three matrices and a question-mark in a 2 × 2 grid. Five response alternatives were also presented. The child was then asked which of the five alternatives fit best to the three other stimuli. The test was terminated if the child gave the wrong answer to four out of five trials in a row.

#### Complex Verbal Working Memory

The sentence completion and recall task from the Sound Information Processing System (SIPS) battery (Wass et al., 2008) was used as a complex verbal working memory measure. The test was adapted from Towse et al. (1998). The task has previously been used for children with CI from the same age group as tested in the current study (Wass et al., 2008). The children heard a recorded sentence, spoken by a female speaker, with the last word missing. The child was then asked to fill in the missing word. A standard instruction was used and the child could practice using two examples before the test started. Those practice trials were also used to adjust the sound level to the needs of the child. The sentence completion and recall task consisted of two sets. Each of those sets consisted of three levels ranging from two to four sentences. After the child listened to all the sentences within one block and filled in the missing last word, she/he was asked to repeat the last words of the sentences. The child got points for every word she/he recalled correctly. The maximum amount of points was 18.

#### Non-verbal Working Memory

As a measure of spatial memory, the Visual Matrix Patterns test from the SIPS battery was used (Wass et al., 2008). In this task, the child saw a 5 × 5 matrix of 25 gray blocks on a computer screen. A pattern of blocks turned black. The child was then asked to recall the pattern by clicking on the respective blocks in the correct order. The difficulty level ranged from one to eight blocks. Each level consisted of three different patterns. The task was aborted if the child was not able to recall at least two of the three patterns for the last two levels. A standard instruction was used. The child got a point for every level in which she/he recalled at least two of the three patterns correctly.

### Procedure

The children were tested one-to-one by a speech and language pathologist or a speech and language pathology student in their last university term. They were tested in a quiet room at school or at home. The test sessions were recorded using a Dictaphone. The order of the tests was randomized. The testing was part of a larger research project and the children were tested on more tasks than are reported here. For the children with CI, a microphone and/or amplifier were used during testing if these resources were available. Otherwise, the children listened to the oral test material using the laptop loudspeakers. The practice trials were used to make sure that the child understood the task and that the sound level was high enough. If the child reported being unable to hear the recordings properly, the sound level was adapted.

### Statistical Analysis

We used R (R Core Team, 2016) with the packages tidyverse (Wickham et al., 2019), effsize (Torchiano, 2020), rstatix (Kassambara, 2020), and car (Fox and Weisberg, 2019) to sort, edit, analyze, and plot our data.

The data were checked for normality. The data for the expressive spoken grammar task were non-normally distributed, and the data for the expressive spoken vocabulary task were normally distributed. The alpha value was set to 0.05.

To analyze if the groups differed in terms of variance for the expressive spoken grammar task, a Fligner-Killeen tests was used. In addition, the range and interquartile range (IQR) were calculated. To analyze the differences between the groups in terms of their expressive spoken grammar ability, Mann-Whitney U tests were calculated. *r* was used as a measure of effect size for this analysis. To analyze if groups differed in terms of variance for the expressive spoken vocabulary task, a Levene’s test was used. To evaluate differences between the groups in terms of their expressive spoken vocabulary, a *t*-test was calculated. Cohen’s *d* was used as a measure of effect size for this analysis.

As the group of children with CI was diverse in terms of age of implantation, correlations were calculated between the age of implantation and both expressive spoken language measures to evaluate the effect of these differences.

## Results

### Expressive Spoken Grammar

**Figure 1:**
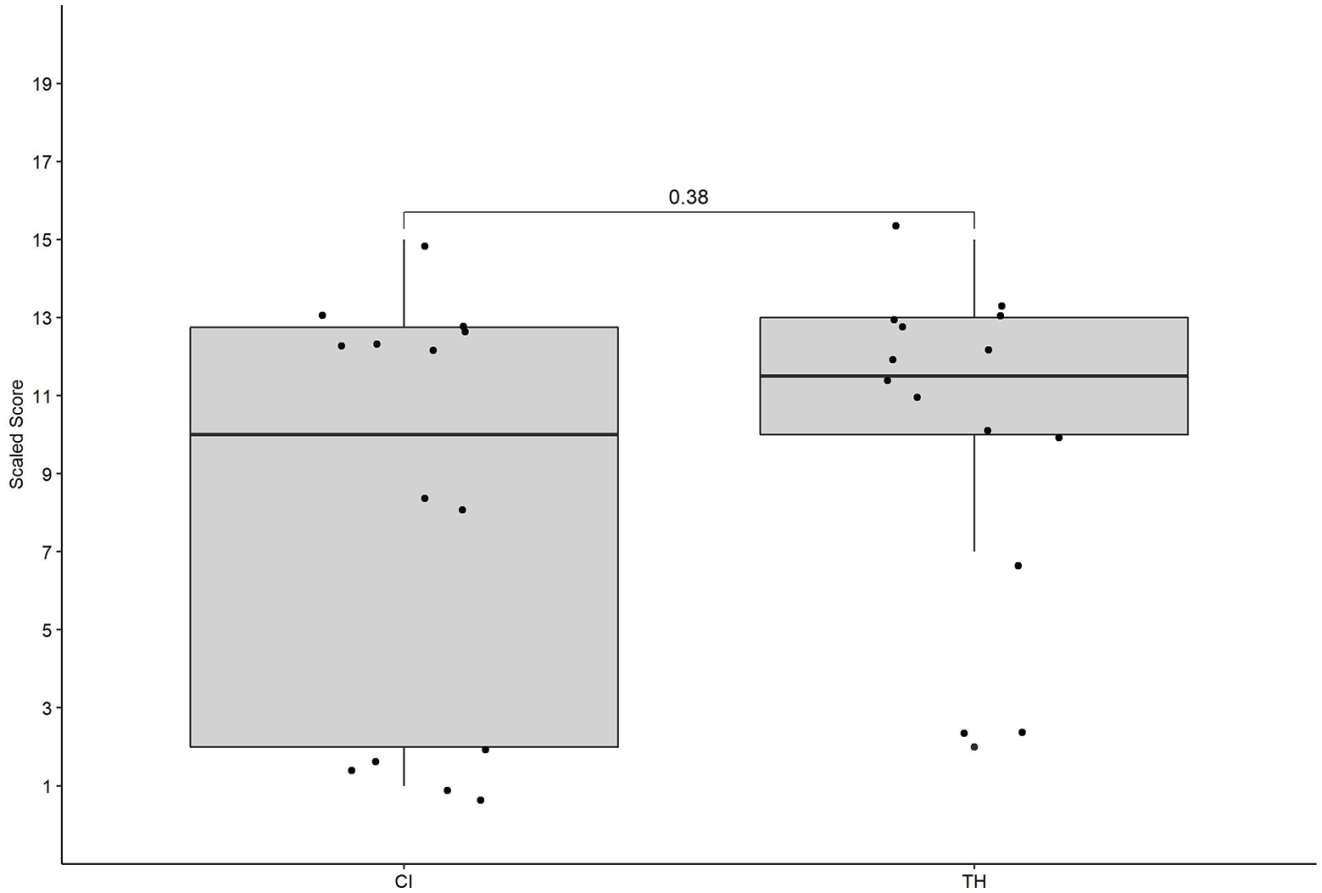
The performance of the children with cochlear implants (CI) and the children with typical hearing (TH) on the expressive spoken grammar task. The individual data as well as the group data and the *p* of the group comparison are presented.

No significant difference in terms of variance was found between the children with CI and the children with TH, *χ*^2^ = 3.198, *p* = 0.07. The range was slightly larger for the children with CI (14) compared to children with TH (13). In addition, a difference in IQR was found between the children with CI (10.8) and the children with TH (3), indicating that the middle 50% of the data were more spread out for the children with CI. No significant difference was found in terms of performance, *U* = 78.5, *p* = 0.376, *r* = 0.172. Group performance is graphically presented in Figure 1. The minimum, maximum, IQR, median, and statistical comparisons are presented in Table 2.

**Table 2:** The minimum, maximum, IQR, and median for the expressive spoken grammar task for the children with CI and children with TH.

Group	Minimum	Maximum	IQR	Median	Comparison variance	Comparison median
CI	1	15	10.8	10	*X*^2^ = 3.198, *p* = 0.07	*U* = 78.5, *p* = 0.376, *r* = 0.172
TH	2	15	3	11.5		

In addition, the statistical comparison of the variance and the median is presented.

### Expressive Spoken Vocabulary

**Figure 2:**
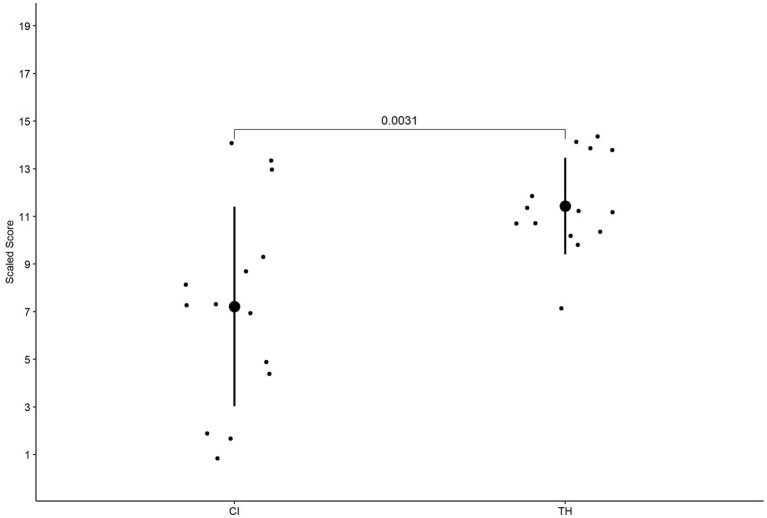
The performance of the children with CI and the children with TH on the expressive spoken vocabulary task. The individual data, as well as the group data (mean and SD) and the *p* of the group comparison, are presented.

A significant difference in terms of variance was found between the children with CI and the children with TH, *F*(1, 26) = 5.192, *p* = 0.031. The range was larger for the children with CI (13) compared to children with TH (7). In addition, a difference in IQR was found between the children with CI (4.75) and the children with TH (3.25). A significant difference was found in terms of performance, *t*(18.767) = −3.387, *p* = 0.003, and *d* = 1.28, with the children with CI performing significantly more poorly than the children with TH. Group performance is graphically presented in Figure 2. The minimum, maximum, IQR, median, and statistical comparisons are presented in Table 3.

**Table 3:** The minimum, maximum, IQR, and mean for the expressive vocabulary task for the children with CI and children with TH.

Group	Minimum	Maximum	IQR	Mean	Comparison variance	Comparison mean
CI	1	14	4.75	7.21 (4.19)	*F*(1, 26) = 5.192, *p* = 0.031	*t*(18.767) = −3.387, *p* = 0.003, *d* = 1.28
TH	7	14	3.25	11.4 (2.03)		

In addition, the statistical comparison of the variance and the mean is presented.

## Discussion

The aim of the current study was to analyze differences in expressive spoken language skills of children with CI and children with TH matched both on non-verbal intelligence and working memory. The results indicated that children with CI perform significantly more poorly on an expressive spoken vocabulary task compared to children with TH but that there is no significant difference between the groups in terms of expressive spoken grammar. In addition, the variance in the groups was found to differ significantly for the expressive spoken vocabulary but not for the expressive spoken grammar task. The IQR was, however, considerably larger for the children with CI compared to the children with TH for the expressive spoken grammar task and there was a tendency toward a significant difference in terms of variance.

The children with CI taking part in the current study were found to not perform significantly, more poorly on a standard expressive spoken grammar task. The effect size was small, indicating only a small difference between the groups. This result is in contrast to previous studies (Inscoe et al., 2009; Boons et al., 2013). An important difference between the current study and the study by Boons et al. (2013) is the matching procedure. Boons et al. (2013) did not match the children on cognitive abilities. They excluded children with a non-verbal IQ below 80 but did not evaluate whether this led to two groups with a comparable average non-verbal IQ and a comparable variance in non-verbal IQ. Part of the difference found by Boons et al. (2013) could, therefore, be explained by differences in cognitive abilities. Cognitive abilities, like non-verbal intelligence (van der Schuit et al., 2011; Cupples et al., 2018) and verbal working memory (Willstedt-Svensson et al., 2004), have been found to be associated with expressive spoken grammar competence, and differences between the groups in these abilities might lead to larger differences in the language domain. However, the influence might as well be the other way around. It has been argued that tasks which encourage verbal rehearsal strategies are influenced by language ability (von Koss Torkildsen et al., 2018). The verbal working memory task used in the current study is such a task. In addition, language ability has also been found to influence the performance on the Matrix task (Dugbartey et al., 1999), which has been used as a non-verbal intelligence measure in the current study. It could therefore be argued that the language ability of the children might influence their performance on the cognitive tasks and therefore no differences have been found for expressive spoken grammar after matching them on non-verbal intelligence and verbal working memory. It could be argued that the test used in the current study underestimates the differences between the groups as it does not control for the complexity of the grammatical structures used by the child or the exact number of errors (Semel et al., 2013). The child receives 0 points for a task as soon as she/he makes more than two errors. This standard scoring might lead to undetected differences in the number of grammatical errors in the two groups. However, Boons et al., 2013 used the same task as used in the current study and found a significant difference between children with CI and children with TH. Furthermore, the Formulate Sentences task is currently the only standardized expressive spoken grammar measure for Swedish.

In accordance with previous research (Boons et al., 2013; Lund, 2016), this study has found a significantly lower performance of children with CI on a standard expressive spoken vocabulary compared to children with TH. Lund (2016) argues that it is hard to catch up with vocabulary skills as vocabulary continues to grow throughout life. This might be an explanation as to why children with CI in the current study performed lower on the expressive spoken vocabulary task even though they showed no significant difference to children with TH in terms of expressive spoken grammar. However, a smaller vocabulary might have large implications for the ability to communicate complex thoughts. Even if a child can use complex sentence structures, those are of no use if they cannot be filled with words. In addition, Castellanos et al. (2016) found that expressive spoken vocabulary skills are an important predictor for general expressive spoken language ability, executive function, and academic skills in children with CI. A delayed expressive spoken vocabulary ability could, therefore, have implications for a child’s school success. However, Lund and Douglas (2016) found that direct, explicit teaching of vocabulary might be the most promising way to increase vocabulary skills in deaf and hard of hearing children. More research is needed to evaluate this kind of language training and its implication on long-term expressive spoken language outcomes in children with CI.

Studies comparing children with CI to children with TH in terms of expressive spoken language seldom statistically evaluate (or perhaps report) differences in variance between the groups (Boons et al., 2013; Hess et al., 2014). Nevertheless, it is often stated that children with CI show a greater variability in terms of spoken language outcome. However, at a certain age, a large variance in language ability is also characteristic of typical development (Kidd et al., 2018). It is, therefore, important to compare children with CI to a well-matched group of children with TH. This makes it possible to evaluate if the spread in terms of spoken language ability is significantly larger for children with CI than would be expected by their variance in cognitive ability. In the current study, it was found that the variance in terms of expressive spoken vocabulary is significantly larger for children with CI even if they are compared to children with TH matched on non-verbal intelligence and working memory. This significant difference was most likely due to a larger range in the performance level of children with CI. The variance in terms of expressive spoken grammar was not found to significantly differ between children with CI and children with TH. However, this might be due to the small sample size as there was a tendency toward a significant difference, and the interquartile range was considerably larger for children with CI. This is most likely due to more children with CI (5) than children with TH (3) performing in the lower range. Boons et al. (2013) also found that children with CI tend to perform at or below the age norm, while children with TH perform at or above the age-norm. Expressive language abilities are important for children, both to take part in social interactions and to actively take part in school classes. Therefore, future studies should investigate what is causing the large spread in expressive spoken language ability of children with CI and the tendency to perform at lower levels than children with TH.

There are several possible explanations as to why the variance in performance was larger for children with CI. It might be the case that additional confounding factors which are not present in the group of children with TH, like age at implantation (Nicholas and Geers, 2006; Leigh et al., 2013; Dettman et al., 2016), time of language deprivation (Hall et al., 2019), hearing thresholds before implantation (Nicholas and Geers, 2006), or schooling (Geers et al., 2003b) led to a larger spread in performance. In addition, the use of an oral language test might have underestimated the expressive language ability of some children with CI, especially those using signs as support or sign-language in addition to oral language. An alternative explanation might be that cognitive factors play a more important role for the expressive spoken language of children with CI. The input provided by a CI is sparser than in TH (Zeng, 2004; Loizou, 2006), thus spoken language might be more cognitively demanding when a CI is used. This is, however, only speculative and studies with larger samples are needed to evaluate the predictors for expressive spoken language in children with CI and children with TH matched on cognitive abilities.

### Limitations of the Study

Research on deaf and hard of hearing children with CI is challenging. The group is small and recruiting a large number of participants especially in countries with small populations is often not possible. The current study is no exception and due to the small sample size, the results presented here must be interpreted with caution. Replications are needed and the combination of results from different labs by means of meta-analysis would be highly recommended. In order to compare predictors for expressive spoken language development for children with CI and children with TH, studies are needed which compare groups closely matched on cognitive abilities. A further limitation of the current study apart from the small sample size is the use of oral outcome measures. Several of the children with CI were reported to use sign-as-support and two of the children with CI were bilingual, using sign-language, and oral language. Their true expressive language ability might be underestimated when using a solely oral task. In addition, it is not clear if the matching for verbal working memory has led to the groups being matched on confounding factors, like speech perception ability. The verbal working memory task used in the current study was a sentence completion and recall task. This type of task is highly dependent on language skills. However, it was possible to assess if the children perceived the sentence correct by judging their responses in completion phase. Children mishearing the sentence would likely fill in a fitting word. The children were tested by experienced test-leaders and those were able to adapt the testing to the needs of the child. In addition, when scoring the test, the children received credits for remembering the words they used in in the completion phase. Therefore, wrong answers in the completion phase did not influence the scores of a child. Using this kind of scoring was intended to reduce the influence of vocabulary knowledge and receptive language ability on the performance of the children on the verbal working memory task.

## Conclusion

The current study found that for children with CI individual differences in expressive spoken language ability are larger than for children with TH matched on non-verbal IQ and working memory ability. The data indicate that more children with CI perform at low levels. The larger spread in performance might be explained by additional confounding factors in the group of children with CI, like age at implantation, or by a greater importance of cognitive abilities for the acquisition of oral language for children with CI. The results from the current study indicate that children with CI perform comparably to children with TH on an expressive spoken grammar task if the groups are matched on cognitive ability. This could be explained both by an influence of verbal working memory and non-verbal intelligence on expressive spoken grammar ability but also by an influence of expressive spoken grammar ability on the two cognitive measures. The direction of the influence should be the focus of further studies. Differences in expressive spoken vocabulary do not seem to be explained by cognitive differences, and other factors like the time of language deprivation might be an important factor for this ability for children with CI.

## Data Availability Statement

The datasets generated for this study will not be made publicly available. It was ensured to the parents in the information letter that no data will be send to anyone not part of the research team. This was also included in the ethics application. Requests to access the datasets should be directed to MS, michaela.socher@liu.se.

## Ethics Statement

The studies involving human participants were reviewed and approved by Research Ethics Review Committee in Linköping. Written informed consent to participate in this study was provided by the participants’ legal guardian/next of kin.

## Author Contributions

The study was prepared and designed by MS, MW, and BL. Analysis and interpretation of result were carried out by mainly MS, RE, and MW. The first draft of the manuscript was written by MS. MS, MW, RE, and BL took part in critical revision of the manuscript. All authors contributed to the article and approved the submitted version.

### Conflict of Interest

The authors declare that the research was conducted in the absence of any commercial or financial relationships that could be construed as a potential conflict of interest.
